# Knowledge, attitudes and practices of young people in Zimbabwe on cervical cancer and HPV, current screening methods and vaccination

**DOI:** 10.1186/s12885-019-6060-z

**Published:** 2019-08-28

**Authors:** Witness Mapanga, Brendan Girdler-Brown, Elvira Singh

**Affiliations:** 1 School of Health Systems and Public Health, Epidemiology & Biostatistics, University of Pretoria, 5-10 H.W. Snyman Building, Pretoria, South Africa; 2 Centre for Health Policy, School of Public Health, University of Witwatersrand, Johannesburg, South Africa; 3 Harare, Zimbabwe; 4 Cancer Epidemiology Research Group, National Cancer Registry, National Health Laboratory Service, Johannesburg, South Africa; 5 Community Medicine Unit, School of Public Health, University of Witwatersrand, Johannesburg, South Africa

**Keywords:** Knowledge, Attitude, Young people, Cervical cancer, Zimbabwe

## Abstract

**Background:**

The rise in cervical cancer trends in the past two decades has coincided with the human immunodeficiency virus (HIV) epidemic especially in the sub-Saharan African region. Young people (15 to 24 years old) are associated with many risk factors such as multiple sexual partners, early sexual debut, and high HIV incidences, which increase the chances of developing cervical cancer. The National Cancer Prevention and Control Strategy for Zimbabwe (2014–2018) highlights that no cancer communication strategy focusing on risk factors as primary cancer prevention. Therefore, the study aims to determine the knowledge, attitude and practices of young people in Zimbabwe on cervical cancer, screening, human papillomavirus (HPV) and vaccination.

**Methods:**

A cross-sectional survey assessing young people’s knowledge, attitude and practices concerning cervical cancer was conducted in five provinces in Zimbabwe. A total of 751 young people were recruited through a three-stage cluster design from high schools and universities. Knowledge, attitudes and practices were assessed using questions based and adapted from the concepts of the Health Belief Model (HBM) and the Cervical Cancer Measuring tool kit-United Kingdom (UK).

**Results:**

Most young people, 87.47% (656/750) claimed to know what the disease called cervical cancer is, with a mean score of 89.98% [95% CI 73.71.11–96.64] between high school and 86.72% [95% CI 83.48–89.40] among university students. There was no significant difference in mean scores between high school and university students (*p* = 0.676). A risk factor knowledge proficiency score of ≥13 out of 26 was achieved in only 13% of the high school respondents and 14% of the university respondents with a broad range of misconceptions about cervical cancer risk factors in both females and males. There was not much difference on comprehensive knowledge of cervical cancer and its risk factors between female and male students, with the difference in knowledge scores among high school (*p* = 0.900) and university (*p* = 0.324) students not statistically significant. In contrast, 43% of respondents heard of cervical cancer screening and prevention, and 47% knew about HPV transmission and prevention. Parents’ educational level, province and smoking, were some of the factors associated with knowledge of and attitude towards cervical among high school and university students.

**Conclusion:**

This study revealed that young people in Zimbabwe have an idea about cervical cancer and the seriousness thereof, but they lack adequate knowledge of risk factors. Cervical cancer education and awareness emphasising causes, risk factors and care-seeking behaviours should be commissioned and strengthen at the community, provincial and national level. Developing a standard cervical cancer primary prevention tool that can be integrated into schools can be a step towards addressing health inequity.

**Electronic supplementary material:**

The online version of this article (10.1186/s12885-019-6060-z) contains supplementary material, which is available to authorized users.

## Background

Engaging in risky sexual behaviour and insufficient knowledge about health issues remain at alarmingly high levels among young people aged between 15 to 24 years old in Africa [1]. This was supported by findings that showed that condom use among the 15 to 24-year-olds in sub-Saharan Africa (SSA) was only at 57% for young men and 37% for young women, which was below the 95% target advocated by United Nations General Assembly Special Session on HIV and AIDS in 2001 [2]. In 2015, 17% of young women aged 15–19 years in Zimbabwe reported having had sex with a man 10 years older than themselves in the past 12 months. Also, HIV prevalence among young people in Zimbabwe increases with age, from around 3% in women aged 15–17 years to 14% among the 23–24-year-olds. Among young men, the HIV prevalence rises from about 2.5% among the 15–17-year-olds to around 6% among the 23–24-year-olds [3]. However, only 64% of young women and 47.5% of young men have ever been tested for HIV [4].

Sexual behaviour of both men and women is a risk factor for cervical cancer. Though they do not develop cervical cancer, assessing knowledge, attitude and practices of young men can be vital if a coordinated inclusive strategy towards prevention of cervical cancer is to be formulated as advocated by World Health Organisation (WHO) in 2009. Also, involving men in cervical cancer initiatives such as the human papillomavirus (HPV) vaccination has a cost-benefit relationship that makes it necessary for them to be incorporated in cervical cancer prevention strategies [5]. Knowledge and awareness of cervical cancer and HPV are consistently low across developing countries and such lack of knowledge provides a challenge to the implementation of cervical cancer programmes and the new mass HPV vaccination drive [6]. Evidence from a developing country among women aged 18 to 44 years old indicated that the majority of women are unfamiliar with cervical cancer, HPV, vaccination and screening and that they face several barriers accessing cervical cancer screening services [7, 8].

Sources of information where people get to know about cervical cancer are still limited in developing countries. For example, a study suggested that vaccinated girls were likely to know about cervical cancer if their mothers had previously been screened for cervical cancer [9]. This finding suggests that there is some sort of passing down of health knowledge within families especially when parents have utilised health services. However, in a country like Zimbabwe where over 80% of rural women had no previous knowledge about cervical cancer [10], such passing down of knowledge from parents to children is likely not to exist. Besides, with the cultural nature in Zimbabwe, young people’s sexuality issues are rarely discussed openly in families [11], making it interesting to ask some questions on how much young people have learnt from their parents about cervical cancer.

On the other hand, recent years have witnessed an increase in risky lifestyle behaviour including early onset of sexual activity, multiple sexual partners and age-disparate relationships among the 15 to 24 year age group, resulting in high HIV incidence and placing young women at risk [3]. HIV incidence for young women between the ages of 15–24 years is reported to be twice higher as compared to young men of the same age-group in Zimbabwe [4]. Thus the need to understand the knowledge, attitude and practices of young people towards cervical cancer.

There is scant evidence about young people’s knowledge and understanding of cervical cancer, risk factors, screening and HPV vaccination in the developing world. Do young people know about cervical cancer? What are their beliefs and attitude towards cervical cancer risk factors, screening and HPV vaccination? Do they know how and where to access cervical cancer services in the country? Therefore, the study aimed to determine the knowledge, attitude and practices of young people in Zimbabwe on cervical cancer, screening, HPV and vaccination so as provide relevant information to cervical cancer stakeholders and programme implementers on how to attract young people in addressing cervical cancer.

## Methods

### Study design, setting and period

This cross-sectional study took place in six high schools and five universities in five provinces in Zimbabwe from August to November 2017. The selected sites were located in urban and rural settings in each of the five provinces.

### Study population

The study population was all young people, 15 to 24 years old attending high school or university in Zimbabwe.

### Sample size determination and sampling method

Power analysis was conducted assuming a power of .80 and an alpha of .05. An average response rate of 85% was factored-in to adjust for sample sizes. A design effect of 3 was also factored-in as an adjustment to the survey sample size, due to the cluster sampling that was involved. Two distinct sample sizes of 531 and 447 were calculated for the 15–19 and 20–24-year-olds, respectively.

The study participants were recruited from six high schools and five universities in five of the ten provinces in Zimbabwe. Two separate samples, high school and university students, were chosen differently. Three-stage cluster sampling was used to select study participants (see Additional file 1). Zimbabwe’s ten provinces were used as the sampling units for the first stage. The ten provinces were written on separate paper sheets and these were put in a box where a lottery method was used to select five provinces. The districts of the selected five provinces were used as the sampling units for the second stage. The same process was used. All the districts in each of the selected five provinces were placed in a box for lottery selection of one district per previously selected province. Universities in each of the five selected provinces and high schools within the five selected districts comprised the third stage of sampling. High schools participants who provided consent were recruited for the study through a modified systematic random sampling of every fifth student. High school class lists were used as sampling frames. There was an automatic inclusion in the study for the universities within the five selected provinces. If a selected province had more than one university, a random selection of one university among the total was carried out (see Additional file 1). Purposive sampling was used to select university participants. This was because it was difficult and challenging in terms of the logistics to disrupt lectures to have all the potential participants in a central place. Sex was not considered as a selection criterion for either high school or university participants.

**Fig. 1:**
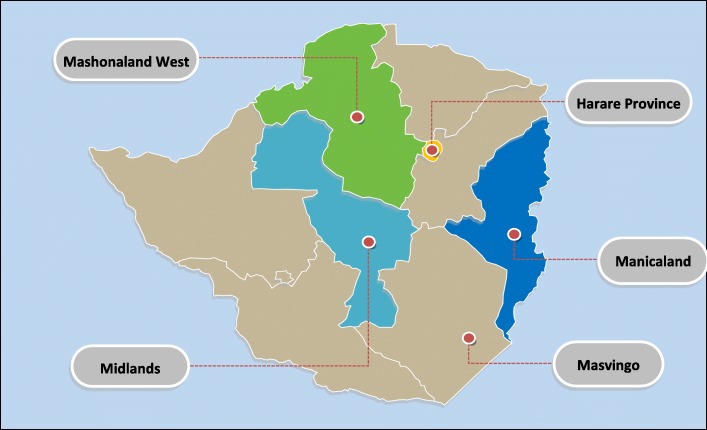
Map of the five selected provinces. Source of the editable map https://yourfreetemplates.com/free-zimbabwe-editable-map/1

The following five provinces, Mashonaland West, Midlands, Masvingo, Manicaland and Harare, were selected for inclusion out of the ten provinces in Zimbabwe (see Fig. 1). Harare has more than one university; therefore, a random selection of one university was carried out as well as a selection of two high schools.

### Data collection

Data were collected from August to November 2017 using a self-administered questionnaire in the presence of the researcher or research assistants. At all levels of data collection and aggregation, there was continuous checking of data quality. The questionnaire’s questions were based and adapted from the concepts of the Health Belief Model (HBM) and the Cervical Cancer Measuring tool kit-United Kingdom (UK) and the content validity was established by giving the questions to experts to assess their relevance in line with the study objectives. After validation, the questionnaire was pilot-tested on 40 randomly selected young people. The questionnaire covered a range of issues including: (1) demographics, (2) cervical cancer knowledge, (3) cervical cancer risk factors, (4) HPV knowledge, (5) cervical cancer screening and vaccination. The HBM dimensions were applied to understand young people’s responses towards cervical cancer (perceived susceptibility, perceived seriousness of the disease), their lifestyle concerning cervical cancer (perceived benefits), health-seeking nature (perceived barriers) and how their demographic characteristics (modifying variables) affect these perceptions.

The total score on knowledge was calculated by combining the scores of the following three sections: knowledge of cervical cancer risk factors including HPV knowledge and vaccination; knowledge of perceived groups at high risk of developing cervical cancer; and knowledge of cervical cancer treatment. The maximum possible score for the knowledge of cervical cancer was 26 and scoring 13 or more was classified as knowing cervical cancer. The total score on positive attitude towards cervical cancer was calculated by combining the scores of the following three sections: perceived attitude towards cervical cancer; perceived feelings towards people with cervical cancer; and perceived health-seeking behaviour. The maximum possible score for a positive attitude towards cervical cancer was 17 and scoring 9 and above were classified as having a positive attitude towards cervical cancer. The practices of young people towards cervical cancer were measured through open-ended questions on their perceived risk of cervical cancer, ways they are preventing cervical cancer and health-seeking behaviours towards screening.

### Ethical considerations

The researcher and his two research assistants received 3 days of training. The training covered among others, study information giving and informed consent process, administration of the questionnaire and general data management processes. Ethical permission to conduct the study was obtained from the Ethics Committee School of Health Systems and Public Health, University of Pretoria, ministries of Health and Child Care, Primary and Secondary Education, and Higher and Tertiary Education in Zimbabwe, and the Medical Research Council of Zimbabwe. Written informed consent from all participants and parents (of those students under the age of 18 years) was sought before data collection.

### Data analysis

Data were analysed using Stata Software Version 14.0. Descriptive statistics for the study samples were calculated without any adjustment for the complex sample design since the aim was simply to describe the samples. However, for analytical hypothesis testing and regression modelling the clustering inherent in the study design was taken into account using Stata’s survey (“svy”) module.

The profile of the respondents was used to identify certain shared or divergent traits. To assess knowledge on cervical cancer, cervical cancer risk factors, cervical cancer screening and HPV vaccination, frequencies and percentages were used to express the results. Cronbach’s alpha was used to measure the internal consistency of the Likert questions used to form construct variables. To determine the factors associated with knowledge of cervical cancer, its risk factors, screening and HPV vaccination, logistic regression models were used. Variables with a *p*-value of 0.25 or under in univariate analyses were unconditionally included in the initial saturated backward stepwise regression model [12]. Following stepwise hierarchical backwards regression modelling, explanatory variables were only removed from the models if the results of a likelihood-ratio (LR) test yielded a *p*-value of greater than 0.283. Results of the association were expressed as adjusted odds ratios with 95% confidence intervals. Post-regression tests were carried out to assess the goodness of fit of the regression model as well as the area under the roc curve [12]. Relationship of association between knowledge and attitude was determined using Chi-squared tests. Significance was assumed at a two-sided value of *p* < 0.05.

To each participant of the selected high school sample, a weight equal to the inverse of the probability of selection was calculated and taken into consideration to obtain estimates of population parameters. The weighting process accounted for the sample selection, important since the initial probabilities of selection were not influenced by population sizes of the sampling units. The weight adjustments coincided with known totals of the high schools, districts and province populations.

## Results

We planned to interview a total of 978 participants, 531 high school and 447 university students. Purposive sampling was used to recruit university students resulting in 513 completing the interview; that is 66 more students interviewed than the initially targeted number. However, some high school students did not get interviewed (in all cases there was no reason given or lack of parental consent). A total of 751 (238 high school and 513 university) students participated in the study. The response rate among the high school children was thus 238/531 = 44.82%.

### Socio-demographic characteristics of intended participants

The majority of the participants were females in both samples. Female students constituted 68.91 and 60.82% of the participants among high school and university samples, respectively (see Table 1). The participants’ ages ranged from 15 to 21 years old among high schools and from 18 to 24 years among university students. Among high school students, those who were 15 (21.43%) and 18 (24.79%) years old, constituted the biggest numbers. Among university participants, 24.76% were 20 years old and only 10.72% were 24 years old.

**Table 1:** Distribution of gender and age of participants

Level of education				Frequency		Percentage (%)							
High school students													
Female				164		68.91							
Male				74		31.09							
University students													
Female				312		60.82							
Male				201		39.18							
High school students	Age	Harare		Manicaland		Mash. West		Masvingo		Midlands		Total	
		Frequency	Percent	Frequency	Percent	Frequency	Percent	Frequency	Percent	Frequency	Percent	Frequency	Percent
	15	22	30.99	0		6	14.29	6	13.64	17	43.59	51	21.43
	16	16	22.54	0		12	28.57	7	15.91	11	28.21	46	19.33
	17	18	25.35	0		12	28.57	7	15.91	4	10.26	41	17.23
	18	15	21.13	21	50.00	7	16.67	11	25.00	5	12.82	59	24.79
	19	0		19	45.24	4	9.52	13	29.55	2	5.13	38	15.97
	20	0		1	2.38	1	2.38	0		0		2	0.84
	21	0		1	2.38	0		0		0		1	0.42
	Total	71	100.00	42	100.00	42	100.00	44	100.00	39	100.00	238	100.00
University students	18	5	3.82	2	3.28	0		1	0.94	1	0.85	9	1.75
	19	32	24.43	19	31.15	6	6.19	3	2.83	15	12.71	75	14.62
	20	32	24.43	19	31.15	24	24.74	19	17.92	33	27.97	127	24.76
	21	31	23.66	8	13.11	24	24.74	22	20.75	25	21.19	110	21.44
	22	15	11.45	5	8.20	19	19.59	19	17.92	22	18.64	80	15.59
	23	8	6.11	4	6.56	10	10.31	20	18.87	15	12.71	57	11.11
	24	8	6.11	4	6.56	14	14.43	22	20.75	7	5.93	55	10.72
	Total	131	100.00	61	100.00	97	100.00>	106	100.00	118	100.00	513	100.00

Most of the participants resided in high-density areas; 55.46% of high school and 50.88% of university students (see Table 2). The majority of both high school (92.44%) and university (93.96%) students described themselves as Christians. Half of the university students, 50.49% (259/513) had ever consumed alcohol as compared to 12.61% (30/230) of high school students. Only 3.70% (19/513) of university students were married with 0.39% (2/513) having been widowed.

**Table 2:** Other socio-demographic characteristics of participants

	Frequency	Percent
High school students		
Province		
Harare	71	29.83
Manicaland	42	17.65
Mashonaland West	42	17.65
Masvingo	44	18.49
Midlands	39	16.39
Residential area		
High density suburb	132	55.46
Low density suburb	53	22.27
Rural area	53	22.27
Religion		
Christianity	220	92.44
Traditional	1	0.42
Apostolic sect	14	5.88
Muslim	2	0.84
None	1	0.42
Ever taken alcohol		
no	208	87.39
Yes	30	12.61
Do you smoke		
No	237	99.58
Yes	1	0.42
Marital status		
Single	238	100.00
University students		
Province		
Harare	131	25.54
Manicaland	61	11.89
Mashonaland West	97	18.91
Masvingo	106	20.66
Midlands	118	23.00
Residential area		
High density suburb	261	50.88
Low density suburb	184	35.87
Rural area	68	13.26
Religion		
Christianity	482	93.96
Traditional	4	0.78
Apostolic sect	13	2.53
Muslim	6	1.17
None	8	1.56
Ever taken alcohol		
no	254	49.51
Yes	259	50.49
Do you smoke		
No	480	93.57
Yes	33	6.43
Paid employment		
No	498	97.08
yes	15	2.92
Marital status		
Single	492	95.91
Married	19	3.70
Widowed	2	0.39

### Knowledge about the disease called cervical cancer

Most young people, 87.47% (656/750) claimed to know what the disease called cervical cancer is, with a mean score of 89.98% [95% CI 73.71.11–96.64] amongst high school and 86.72% [95% CI 83.48–89.40] among university students. There was no significant difference in mean scores between high school and university students (*p* = 0.676).

### Knowledge score for cervical cancer and its risk factors among high school and university students

There was not much difference in a comprehensive knowledge of cervical cancer and its risk factors, based on the calculated overall scores for both high school and university students (see Table 3). Only 12.80% (21/164) of high school female students managed a knowledge score about cervical cancer and its risk factors of 13 and above as compared to 13.51% (10/74) of high school male students. However, the difference in knowledge scores among high school students was not statistically significant (*p* = 0.900). This trend was also found among university students, with only 1.28% (4/312) of university female students scoring a knowledge score about cervical cancer and its risk factors of 13 and above as compared to 2.49% (5/201) of university male students. The difference in cervical cancer knowledge among university students was also not statistically significant (*p* = 0.324).

**Table 3:** Cervical cancer knowledge scores among high school and university students

Cervical cancer knowledge score	Female	Male	Total
Total scores (out of 26)	Frequency (%)	Frequency (%)	Frequency (%)
Cervical cancer knowledge scores for high school students*			
0	27(16.46)	9(12.16)	36(15.13)
1	18(10.98)	11(14.86)	29(12.18)
2	5(3.05)	3(4.05)	8(3.36)
3	4(2.44)	4(5.41)	8(3.36)
4	10(6.10)	4(5.41)	14(5.88)
5	9(5.49)	8(10.81)	17(7.14)
6	10(6.10)	1(1.35)	11(4.62)
7	18(10.98)	7(9.46)	25(10.50)
8	12(7.32)	4(5.41)	16(6.72)
9	8(4.88)	3(4.05)	11(5.00)
10	7(4.27)	5(6.76)	12(5.04)
11	6(3.66)	2(2.70)	8(3.36)
12	9(5.49)	3(4.05)	12(5.04)
13	8(4.88)	4(5.41)	12(5.04)
14	4(2.44)	4(5.41)	8(3.36)
15	5(3.05)	1(1.35)	6(2.52)
16	4(2.44)	1(1.35)	5(2.10)
Total (%)	164 (100)	74(100)	238(100)
Cervical cancer knowledge scores for university students**			
0	48(15.38)	39(19.40)	87(16.96)
1	48(15.38)	33(16.42)	81(15.79)
2	38(12.18)	15(7.46)	53(10.33)
3	16(5.13)	10(4.98)	26(5.07)
4	19(6.09)	18(8.96)	37(7.21)
5	16(5.13)	17(8.46)	33(6.43)
6	26(8.33)	13(6.47)	39(7.60)
7	20(6.41)	11(5.47)	31(6.04)
8	34(10.90)	15(7.46)	49(9.55)
9	16(5.13)	8(3.98)	24(4.68)
10	17(5.45)	5(2.49)	22(4.29)
11	8(2.56)	10(4.98)	18(3.51)
12	2(0.64)	2(1.00)	4(0.78)
13	0(0)	1(0.50)	1(0.19)
14	1(0.32)	0(0)	1(0.19)
15	0(0)	1(0.50)	1(0.19)
16	0(0)	1(0.50)	1(0.19)
17	1(0.32)	0(0)	1(0.19)
18	1(0.32)	0(0)	1(0.19)
20	1(0.32)	2(1.00)	3(1.00)
Total (%)	312(100)	201(100)	513(100)

*Chi-2 test, Pearson: Designed-based F(1, 5) = 0.0176, p = 0.900

**Fisher’s exact two-tailed p-value = 0.324

Overall, only 43.14% (324/751) had ever heard of cervical cancer prevention or screening and 53.0% (398/751) did not know about HPV, how it is transmitted or prevented. Some of the students indicated that food, having sex with an uncircumcised male partner, smoking, and use of detergents such as bathing soaps and hair removers, are some of the factors contributing to the development of cervical cancer. These misconceptions were among both females and males as illustrated by responses from the participants:

23-year-old university female student suggested; "***I am no longer using bathing soap on my reproductive organ because it contributes to the development of cervical cancer***";

Whilst another 21-year-old female university student suggested, "***having sex with an uncircumcised male partner is dangerous and I wish all men will answer the call to be circumcised so that women will not have to worry about cervical cancer***".

### Factors associated with knowledge of cervical cancer and its risk factors among high school and university students

Multiple variable logistic regression modelling was used to determine the adjusted association between knowledge of cervical cancer and the following factors: age, gender, residence, drinking alcohol, smoking, parents’ education and province. Since 92.44% (220/238) of high school students and 93.96% (482/513) of university students reported religion to be Christianity, we decided not to include religion in the regression modelling. On being predictors of knowledge of cervical cancer, most of these socio-demographic characteristics were not statistically significant.

High school students with parents educated up to O-levels (OR = 2.5; 95% CI = 1.28–4.93) and a qualification below degree (OR = 3.13; 95% CI = 1.15–8.49), were almost 3 times more likely to have higher knowledge scores about cervical cancer as compared to high school students with parents with a university degree or a primary level education (see Table 4). In addition, high school students in Mashonaland West (OR = 2.77; 95% CI = 1.60–4.80) and Midlands (OR = 1.77; 95% CI = 1.06–2.95) provinces were 2 to 3 times more likely to have higher knowledge scores about cervical cancer as compared to high school students in Harare province (see Table 4).

**Table 4:** Factors associated with knowledge of cervical cancer among high school students

Factors associated with knowledge of cervical cancer among high school students							
High school students*		Univariate model		Adjusted Odds Ratio (AORs)			
Main variable		Odds Ratio	p	AOR	p	95% Conf. Interval	
parents education	Reference grp (University degree**)						
	O-levels	2.86	0.007	2.51	0.017	1.28	4.92
	Qualification below degree	3.01	0.029	3.13	0.033	1.15	8.49
	A-levels	3.18	0.048	3.01	0.069	0.88	10.25
	No formal education	3.20	0.351	3.00	0.406	0.13	68.02
Province	Reference grp (Harare)						
	Mashonaland West	2.28	0.019	2.56	0.005	1.59	4.80
	Midlands	1.76	0.052	1.64	0.034	1.06	2.95
	Manicaland	1.31	0.280	1.63	0.094	0.89	2.98
	Masvingo	1.24	0.374	1.45	0.127	0.86	2.43
Residence	Reference grp (High-density)						
	Rural	1.36	0.402				
	Low-density	0.89	0.565				
age***		1.21	0.494				
drinking alcohol		0.72	0.670				
Gender		1.06	0.900				

*Clustering inherent in the study design was taken into account and regression was done using Stata’s survey (“svy”) module for high school students

**Primary level was combined with University degree since there was no difference between the two levels of education

***Age as a continuous variable is liner in relation to the logit (Box-Tidwell test p-value = 0.735)

*Post regression test, using the Pearson’s goodness-of-fit test was carried out without factoring the complex sample design- Pearson’s GOF p-value = 0.430*

Among the university students, those who smoke were almost 8 times likely to have higher knowledge scores about cervical cancer as compared to those who did not smoke (OR = 7.80; 95% CI = 1.29–47.21). Also, university students in Harare and Mashonaland West were more likely to have higher knowledge scores about cervical cancer as compared to the students in the Midlands, Masvingo and Manicaland provinces. However, these observed differences among provinces were not statistically significant (see Table 5).

**Table 5:** Factors associated with knowledge of cervical cancer among university students

Factors associated with knowledge of cervical cancer among university students							
University students		Univariate model		Adjusted Odds Ratio (AORs) Final model			
Variable		Odds Ratio	p	AOR	p	95% Conf. Interval	
Smoker		4.36	0.074	7.80	0.025	1.29	47.21
Residence	Reference grp (High-density)						
	Low-density	4.37	0.073	4.25	0.085	0.82	22.02
	Rural	1.93	0.593	2.12	0.554	0.18	25.47
Province	Reference grp (Harare*)						
	Midlands	0.18	0.113	0.13	0.079	0.01	1.26
	Masvingo	0.20	0.137	0.17	0.122	0.02	1.62
	Manicaland	0.35	0.332	0.28	0.250	0.03	2.46
age**		0.79	0.300				
drinking alcohol		0.48	0.309				
Gender		1.96	0.319				

*Mashonaland West was combined with Harare since there was no difference between the two provinces

**Age as a continuous variable is liner in relation to the logit (Box-Tidwell test p-value = 0.241)

*Post regression tests were conducted to check the model fit. Area under the ROC curve = 0.70 for the final model; Hosmer and Lemeshow Goodness of fit test p-values = 0.09; 0.12 and 0.17 respectively (8, 10 and 12 groups)*

### Cervical cancer attitudes and care-seeking behaviour

Majority of the participants, 94.27% (708/751), acknowledged that young people should be concerned about cervical cancer, with a mean score of 90.30% [95% CI = 85.08–92.59] among high school students and 96.20% [95% CI = 94.75–97.63] among university students. The mean concern for cervical cancer score was not statistical significance (*p* = 0.062) between high school and university students.

### Positive attitude towards cervical cancer scores among high school and university students

There was not much difference in a positive attitude towards cervical cancer, based on the calculated overall scores for both high school and university students (see Table 6). Almost half, 48.17% (79/164) of high school female students managed a positive attitude score towards cervical cancer of 9 and above as compared to 60.81% (45/74) of high school male students. This difference in positive attitude scores towards cervical cancer among the high school students was statistically significant (*p* = 0.018). Among university students, 27.88% (87/312) of university female students had a positive attitude score towards cervical cancer of 9 and above as compared to 31.34% (63/201) of university male students. The difference in positive attitude scores among university students was however not statistically significant (*p* = 0.427).

**Table 6:** Positive attitude towards cervical cancer scores among participants

Attitude towards cervical cancer score	Female	Male	Total
Total scores (out of 17)	Frequency (%)	Frequency (%)	Frequency (%)
Attitude towards cervical cancer scores for high school students*			
0	1(0.61)	0(0)	1(0.42)
1	4(2.44)	2(2.70)	6(2.52)
2	36(21.95)	12(16.22)	48(20.17)
3	13(7.93)	4(5.41)	17(7.14)
4	4(2.44)	2(2.70)	6(2.52)
5	7(4.27)	0(0)	7(2.94)
6	9(5.49)	1(1.35)	10(4.20)
7	6(3.66)	4(5.41)	10(4.20)
8	5(3.05)	4(5.41)	9(3.78)
9	9(5.49)	7(9.46)	16(6.72)
10	8(4.88)	9(12.16)	17(7.14)
11	9(5.49)	7(9.46)	16(6.72)
12	18(10.98)	7(9.46)	25(10.50)
13	22(13.41)	8(10.81)	30(12.61)
14	6(3.66)	5(6.76)	11(4.62)
15	5(3.05)	0(0)	5(2.10)
16	2(1.22)	1(1.35)	3(1.26)
17	0(0)	1(1.35)	1(0.42)
Total	164(100)	74(100)	238(100)
Attitude towards cervical cancer scores for university students**			
0	1(0.32)	0(0)	1(0.19)
1	14(4.49)	9(4.48)	23(4.48)
2	88(28.21)	51(25.37)	139(27.10)
3	33(10.58)	25(12.44)	58(11.31)
4	30(9.62)	16(7.96)	46(8.97)
5	22(7.05)	15(7.46)	37(7.21)
6	13(4.17)	9(4.48)	22(4.29)
7	11(3.53)	7(3.48)	18(3.51)
8	13(4.17)	6(2.99)	19(3.70)
9	5(1.60)	4(1.99)	9(1.75)
10	11(3.53)	4(1.99)	15(2.92)
11	11(3.53)	14(6.97)	25(4.87)
12	12(3.85)	9(4.48)	21(4.09)
13	15(4.81)	9(4.48)	24(4.68)
14	14(4.49)	9(4.48)	23(4.68)
15	12(3.85)	5(2.49)	17(3.31)
16	4(1.28)	6(2.99)	10(1.95)
17	3(0.96)	3(1.49)	6(1.17)
Total	312(100)	201(100)	513(100)

*Chi-2 test, Pearson: Designed-based F(1, 5) = 11.95, p = 0.018

**Fisher’s exact two-tailed p-value = 0.427

When the respondents were asked on the perceived risk of them or their girlfriend or wife (in the case of male respondents) developing cervical cancer, 45.34% (258/569) indicated no perceived risk. Some of the reasons that prompted the no perceived risk were as follows: not being an alcohol drinker or smoker, not using contraceptive pill, having been circumcised or having a circumcised partner, going for regular medical check-ups, being faithful to my partner, not HIV positive, by praying and not being a commercial sex worker.

"***I am not worried about cervical cancer neither is my girlfriend because I am circumcised and we are both faithful***", wrote 23-year-old male university student.

Whilst a 24-year-old female university student wrote, "***commercial sex workers and those who drink alcohol or use drugs are the ones that are as likely to develop cervical cancer not me since I am a Christian***".

Some of the respondents (both males and females) indicated that it is solely the responsibility of those ‘who are likely to develop cervical cancer to seek for cervical cancer prevention’.

"***If I am not mistaken, cervical cancer is a women's disease, so women should be the ones to be responsible and take good care of their health***", suggested 18-year-old male high school student.

Other respondents believed that women who develop cervical cancer are of ‘loose morals’ and ‘ignorant’.

"***I am very particular when it comes to my health; I go for regular check-ups. Women who develop cervical cancer are ignorant***", wrote a 23-year-old female university student.

When asked on what worries them most about cervical cancer, the students indicated that cervical cancer is associated with dying; the expensive nature of the treatment; the stigma that society attaches to cervical cancer; failure to conceive; divorce; the suffering and pain that the patient and their families undergo.

"***What worries me most about cervical cancer is that the patient will obviously die because the cure is expensive***", suggested 15-year-old female high school student;

Whilst a 21-year-old male university student wrote, "***what worries me most is if my girlfriend or wife is to have cervical cancer then I will not have sex and she will not be able to bear children for me. That can be a recipe for separation***".

### Factors associated with a positive attitude towards cervical cancer among high school and university students

Multiple variable logistic regression modelling was used to determine the adjusted association between positive attitude towards cervical cancer and the following factors; age, gender, residence, drinking alcohol, smoking, parents’ education and province. Religion was not included in the regression modelling. Almost no socio-demographic characteristics were statistically associated with a positive attitude towards cervical among high school and university students. High school students with parents educated up to primary level were 84% more likely not to have a positive attitude towards cervical cancer (OR = 0.16; 95% CI = 0.06–0.48) as compared to high school students with parents with a university degree (see Table 7).

**Table 7:** Factors associated with a positive attitude towards cervical cancer among high schools students

Factors associated with positive attitude towards cervical cancer among high school students							
High school students*		Univariate model		Adjusted Odds Ratio (AORs)			
Main variable		Odds Ratio	p	AOR	p	95% Conf. Interval	
age**		1.43	0.010	1.36	0.060	0.98	1.89
Gender		1.65	0.018	2.11	0.057	0.97	4.63
Province	Reference grp (Harare)						
	Manicaland	1.81	0.027	0.82	0.474	0.42	1.59
	Mashonaland West	1.64	0.029	1.67	0.025	1.10	2.54
	Masvingo	1.78	0.030	1.32	0.176	0.84	2.08
	Midlands	0.77	0.231	0.71	0.154	0.41	1.20
parents education	Reference grp (University degree)						
	Primary level	0.14	0.002	0.16	0.007	0.06	0.48
	No formal education	0.24	0.158	0.15	0.225	0.01	5.05
	A-levels	0.58	0.355	0.75	0.663	0.15	3.80
	Qualification below degree	1.19	0.709	1.32	0.561	0.42	4.14
	O-levels	0.91	0.783	0.91	0.815	0.34	2.42
drinking alcohol		0.76	0.566				
Residence	Reference grp (High density)						
	Low density	1.17	0.633				
	Rural	0.93	0.813				

*Clustering inherent in the study design was taken into account and regression was done using Stata’s survey (“svy”) module for high school students

**Age as a continuous variable is liner in relation to the logit (Box-Tidwell test p-value = 0.512)

*Post regression test, using the Pearson’s goodness-of-fit test was carried out without factoring the complex sample design- Pearson’s GOF p-value = 0.182*

Among the university students, those in Harare were more likely to have higher positive attitude scores towards cervical cancer as compared to the students in the Midlands, Mashonaland West, Masvingo and Manicaland provinces. For example, university students in Midlands were 55% more likely not to have a positive attitude towards cervical cancer as compared to students in Harare (OR = 0.45; 95% CI = 0.25–0.81) and this difference was statistically significant (*p* = 0.007). University students with parents, who had a qualification below university degree, A-levels, O-levels or no formal education, were more likely to have a higher positive attitude towards cervical cancer as compared to students with parents with a university degree. However, these observed differences were not statistically significant (see Table 8).

**Table 8:** Factors associated with a positive attitude towards cervical cancer among university students

Factors associated with positive attitude towards cervical cancer among university students							
University students		Univariate model		Adjusted Odds Ratio (AORs)			
Variable		Odds Ratio	p	AOR	p	95% Conf. Interval	
Province	Reference grp (Harare)						
	Midlands	0.46	0.007	0.45	0.007	0.25	0.81
	Mashonaland West	0.65	0.148	0.65	0.142	0.36	1.16
	Masvingo	0.81	0.443	0.80	0.428	0.46	1.39
	Manicaland	0.87	0.676	0.92	0.808	0.48	1.78
parents education	Reference grp (University degree)						
	Qualification below degree	1.59	0.057	1.65	0.054	0.99	2.64
	No formal education	2.01	0.126	1.94	0.151	0.79	4.82
	A-levels	1.45	0.346	1.52	0.299	0.69	3.33
	Primary level	0.65	0.582	0.66	0.604	0.14	3.16
	O-levels	1.16	0.593	1.19	0.538	0.68	2.10
smoker		0.63	0.298				
Gender		1.18	0.401				
drinking alcohol		1.09	0.660				
residence	Reference grp (High-density)						
	Low-density	1.06	0.792				
	Rural	1.03	0.913				
age**		1.00	0.948				

**Age as a continuous variable is liner in relation to the logit (Box-Tidwell test p-value = 0.635)

*Area under the ROC curve = 0.61 for the final model; Hosmer and Lemeshow Goodness of fit test p-values = 0.34; 0.51 and 0.30 respectively (8, 10 and 12 groups)*

### Cervical cancer awareness and need for more information among high school and university students

A quarter of the young people in this study reported feeling well informed about cervical cancer with mean scores of 24.43% [95% CI = 17.14–32.92] and 26.12% [95% CI 22.32–29.92%] among high school and university students, respectively. There was no significance (*p* = 0.586) between the two mean scores of feeling well informed about cervical cancer. However, some of the young people who claimed to feel well informed about cervical cancer, also wished for more cervical cancer information, a mean score of 98.09% [95% CI = 92.97–99.50] among high school students and 96.30% [95% CI = 93.66–97.93%] among university students. The mean scores were also not significant (*p* = 0.196) between the two groups.

### Factors associated with the need for more cervical cancer information among high school and university students

Logistic regression modelling failed to uncover any meaningful relationships between the measured potential explanatory variables and the perceived need for more cervical cancer information among high school and university students. The logistic regression models contained no statistically significant explanatory variables and areas under the receiver operating characteristic (ROC) curve were all less than 0.35. The model F-tests were also non-significant.

## Discussion

For this investigation, 751 out of the projected 978 individuals were recruited using a three-stage cluster design from both high school and universities. This study demonstrated that 87% of the young people in Zimbabwe aged 15 to 24 years who participated in this study knew what cervical cancer is. This rate is significantly larger than the findings reported in Johannesburg, South Africa suggesting that a majority of women 18 to 44 are unfamiliar with cervical cancer [7], and that reported in Zimbabwe showing that over 80% of rural women had no previous knowledge of cervical cancer [11]. This finding is even though the National Cancer Prevention and Control Strategy for Zimbabwe 2013–2017, which encompassed cervical cancer prevention and management, was not fully implemented due to the inadequacy of cancer legislation and resource constraints.

This cross-sectional survey is the first one to be conducted among young people aged 15 to 24 in Zimbabwe on knowledge, attitude and practices towards cervical cancer. This study has shown that there is insufficient knowledge about sexual reproductive health including cervical cancer among young people. Young people in Zimbabwe have a general idea about cervical cancer and the seriousness thereof, but they lack adequate knowledge of risk factors and information on where to access cervical cancer services. A risk factor knowledge proficiency score of ≥13 out of 26 was achieved in only 13% of the high school respondents and 14% of the university respondents with a broad range of misconceptions about cervical cancer risk factors in both females and males. Having heard about cervical cancer and considering it to be serious does not necessarily correspond to young people having a correct understating of the disease and its associated risk factors. There were no significant differences in knowledge of cervical cancer and its risk factors between high school and university students or rural and urban students and this might indicate lack of cancer education or awareness at a national level. Over 85% of both high school and university students did not have enough knowledge about cervical cancer or its risk factors. This lack of knowledge is similar to what was found among a study that assessed women’s knowledge, attitude and practices towards cervical cancer screening in Zimbabwe [10].

The current cervical cancer screening strategies especially in Zimbabwe target women older than 21 years old [13] but this study has shown that the knowledge of the screening services and their availability is very low even among young women between the ages of 21 to 24 years. This finding was similar to what was found in Cambodia, where women aged 20–69 years had a low awareness of cervical cancer screening services, which led to underutilisation of these services [14]. Generally, young people were unable to mention cervical cancer risk factors or know about cervical cancer screening and where it is offered. There was wide ignorance among the respondents on cervical cancer risk factors, with a lot of myths and misconceptions. These findings point to the need for a cervical cancer primary communication strategy or tool targeting young people to improve their health education. Besides, considering the long incubation period for someone to develop cervical cancer, it might be argued that targeting young people in cervical cancer prevention, by screening from the age of 15 and after sexual debut, in light of HIV incidence among them, might be beneficial and cost-effective shortly.

Zimbabwe does not have a cancer primary prevention strategy that focuses on cancer risk factors [13]. The HBM and socio-ecological model (SEM) believe that increasing knowledge and awareness of a disease, in this case, cervical cancer, may play a role in improving healthcare-seeking behaviour among young people towards cervical cancer prevention services [15–17]. Based on the HBM and SEM, lack of information and knowledge about cervical cancer, especially as found in this study, are some of the reasons that contribute to underutilisation of screening services, late diagnosis of the condition, and a high mortality rate [18, 19]. Young people in this study (including the young men’s perception towards their girlfriends and wives) did not feel susceptible to cervical cancer because of various reasons ranging from being faithful, to having being circumcised and not using detergents that are believed to cause cancer. This belief of lack of susceptibility towards cervical cancer by young people is almost similar to what was found in other studies among women [20, 21]. Also, there is a lack of cervical cancer prevention policies even in light of the newly launched Mass HPV Vaccination Programme for young girls in Zimbabwe. These legislation and policy challenges might be contributing to the fragmentation of cervical cancer service provision and failure to prevent ‘silo’ operation among different partners within the sexual reproductive health management space. Such legislation and policy challenges have been cited as barriers in most developing countries and solving them requires information as reported in this study’s findings [22].

Lastly, Zimbabwe remains both a patriarchal and conservative society and most men continue to make decisions on the health of women, from providing for money for hospital fees to deciding if women must seek medical attention. This study highlights the ignorance that young people, especially young men, have on cervical cancer and its screening. Men continue to be sidelined in health prevention programmes and this has fueled their passive nature towards health-seeking behaviour, as some responses from young men in this study have shown [23, 24]. Conservatively, even when parents are informed or educated, the culture of sharing their knowledge with their children is not often practiced. The findings of this study have shown that parents educational level does not correlate with young people’s knowledge of cervical cancer, with those with parents with university degrees having low knowledge as compared to some with parents who have secondary education. This also means parental awareness and knowledge might be low as reported in other countries [25].

### Study limitations

This study had several limitations. The convenient sampling, which was used for university students, made it difficult to generalise their sentiments for the whole country. Also, those who participated in this study might have been different from those who could not participate because they were attending lectures or writing examinations. The research findings might have underestimated the extent of lack of knowledge, attitude and practices of young people towards cervical cancer due to the non-response rate of high school students. The response rate in the high school students was almost 45% and the inability to obtain parental consent accounted for 6% of this low response. The 55% non-response rate among high school students may reflect a decrease in statistical power and this might potentially have undermined the generalisability of the reported results to the larger target audience. The university cohort was oversampled with 531 of a projected 513 sample size goal. This also might have introduced some bias in overestimating the reported results among university students.

## Conclusion

In as much as young people in Zimbabwe have an idea about cervical cancer and the seriousness thereof; they lack adequate knowledge on risk factors as indicated by the broad range of misconceptions. Cervical cancer education and awareness emphasising causes, risk factors and care-seeking behaviours should be commissioned and strengthened at the community, provincial and national level. Developing a standard cervical cancer primary prevention tool that can be integrated into schools can be a step towards addressing this health inequity. This standard cervical cancer primary prevention tool should be guided by the WHO-coordinated strategy towards the prevention of cervical cancer, which sought to include both men and women in cervical cancer initiatives [26]. Involving men in cervical cancer prevention strategies is key to reinforcing the drive to enhance the disease’s management and this has a cost-benefit relationship [27]. Zimbabwe can develop and ring-fence her cervical cancer prevention and management plans by developing a policy in line with WHO coordinated strategy. The policy might need to incorporate primary and community cervical cancer prevention and management initiatives pool together all public and private cervical cancer providers and support a financing system that can provide access to affordable, quality cervical cancer services.

## Additional file

Additional file 1: Three-stage cluster sampling of participants. (DOCX 118 kb)

## Data Availability

The authors declare that all data supporting the findings of this study are available within the article. The dataset was generated and analysed during the current study.

## Abbreviations

AAU: Association of African Universities
HBM: Health Belief Model
HIV: Human immunodeficiency virus
HPV: Human papillomavirus
LR: Likelihood-ratio
MRCZ: Medical Research Council of Zimbabwe
ROC: Receiver operating characteristic
SEM: Socio-ecological model
SSA: sub-Saharan Africa
UK: United Kingdom
WHO: World Health Organisation
