# Supplementary material for: Knowledge, attitudes and practices of young people in Zimbabwe on cervical cancer and HPV, current screening methods and vaccination
Source: BMC Cancer. 2019 Aug 28;19:845. doi: 10.1186/s12885-019-6060-z (PMC6712720; doi:10.1186/s12885-019-6060-z)
Supplement: Supplementary file 1 — Three-stage cluster sampling of participants. (DOCX 118 kb) [file 12885_2019_6060_MOESM1_ESM.docx]

**Additional file 1**

Zimbabwe

The names of the 10 provinces were placed in a box for withdrawal purpose

Bulawayo

Harare

Manicaland

Mash. East

Midlands

Mat. South

Mat. North

Masvingo

Mash. Central

Mash. West

High School Students

University Students

First sampling: Selection of 5 provinces using lottery method

Uni 1

Uni 2

Uni 3

Uni 3

Uni 4

Uni 6

Uni 7

Prov. 5

High S6

High S5

High S4

High S1

High S2

Prov. 4

Prov. 1

Prov. 2

Prov. 3

Dist. A

Dist. H

Dist. M

Dist. S

High S3

Uni 8

Automatic selection of the universities

**Province with more than 1 university**

Random selection of 1 university

Purposive selection of eligible participants

Selection of eligible participants using systematic random sampling

Second sampling: The name of all districts in a province were written and placed in a box. Selection of 1 district per province using lottery method – making total of 5 districts

The name of all high schools in a district were written and placed in a box for lottery selection

Dist. Z
